# Guarana Provides Additional Stimulation over Caffeine Alone in the Planarian Model

**DOI:** 10.1371/journal.pone.0123310

**Published:** 2015-04-16

**Authors:** Dimitrios Moustakas, Michael Mezzio, Branden R. Rodriguez, Mic Andre Constable, Margaret E. Mulligan, Evelyn B. Voura

**Affiliations:** 1 Department of Biology, Colgate University, Hamilton, New York, United States of America; 2 Department of Math and Science, Dominican College, Orangeburg, New York, United States of America; 3 New York College of Podiatric Medicine, New York, New York, United States of America; University of Lancaster, UNITED KINGDOM

## Abstract

The stimulant effect of energy drinks is primarily attributed to the caffeine they contain. Many energy drinks also contain other ingredients that might enhance the tonic effects of these caffeinated beverages. One of these additives is guarana. Guarana is a climbing plant native to the Amazon whose seeds contain approximately four times the amount of caffeine found in coffee beans. The mix of other natural chemicals contained in guarana seeds is thought to heighten the stimulant effects of guarana over caffeine alone. Yet, despite the growing use of guarana as an additive in energy drinks, and a burgeoning market for it as a nutritional supplement, the science examining guarana and how it affects other dietary ingredients is lacking. To appreciate the stimulant effects of guarana and other natural products, a straightforward model to investigate their physiological properties is needed. The planarian provides such a system. The locomotor activity and convulsive response of planarians with substance exposure has been shown to provide an excellent system to measure the effects of drug stimulation, addiction and withdrawal. To gauge the stimulant effects of guarana we studied how it altered the locomotor activity of the planarian species *Dugesia tigrina*. We report evidence that guarana seeds provide additional stimulation over caffeine alone, and document the changes to this stimulation in the context of both caffeine and glucose.

## Introduction

Caffeine is considered the major stimulatory constituent of energy drinks while sugars and other substances such as guarana, taurine, and ginseng are added, often with combinations of vitamins, to formulate different blends [1, 2]. Energy drinks have become an accepted means to increase cognitive ability, memory, alertness, physical performance and cardiovascular output [3–10]. But while energy drink use continues to rise, concerns have been raised about their high caffeine content and with it, the risks associated with their consumption particularly by young people [1, 8, 11–15]. The use of guarana and other plant-based materials can further increase the caffeine content and general stimulant properties offered by these beverages, but because they are considered herbal supplements, these plant-based additives are not subject to the same reporting requirements as are sugars and caffeine. Consequently, the concentration of these supplements, let alone what they might contain, are typically not reported in energy drink formulations. This lack of understanding brings into question how the combination of the resulting ingredients might work together to affect the health of those consuming the foods that contain them [2, 12–14, 16–18].

Guarana (*Paullinia cupana*) is a species of climbing plant native to the Amazon that is known as an antioxidant, traditional medicinal, and an effective stimulant [19–25]. Recent work also examined the use of guarana to counter fatigue and depression associated with cancer treatment [26–28]. The main component of guarana attributed to these beneficial properties is caffeine, which, depending on how the extract is prepared, can be more than four times the amount found in coffee beans [19, 29]. In addition, other components of guarana seeds are also thought to provide extra stimulant effects above those of caffeine alone [19–23, 30]. As such, herbal products such as guarana offer an attractive additive to the manufacturers of many popular energy drink formulations, despite the lack of research focused on the associative properties of plant-based extracts with other dietary supplements, drugs and stimulants, [29].

To assess the role of guarana as a stimulant in comparison to caffeine alone, we developed a simple, easily controllable system using the planarian, *Dugesia tigrina*, a free-living aquatic flatworm, as a model organism. Planarians are most often used in studies focused on tissue regeneration and repair due to their exceptional regenerative capacity [31]. Planarians also lend themselves well to stimulant studies they because they possess a basic bilateral symmetry and corresponding central nervous system that uses neurotransmitter systems comparable to those found in mammals [32–39]. Planarians prefer darkness and use photoreceptors to detect and react to light. As such, planarians are capable of maze learning and can develop a conditioned response to light that can be implemented in behavioral studies including those subject to drug modification [40, 41]. Planarian motility assays have also been developed for studies examining substance addiction and withdrawal [42–53]. Because the planarian motility assay offers a simple, easily controlled system to monitor the effect of stimuli, we adopted the assay for use in our study of guarana. Previous studies also indicated that caffeine does not cause a significant increase in planarian activity [47, 50]. This observation provided an added incentive to use the planarian for our work in that it provides an opportune background to examine the tonic effects of guarana separate from those provided by caffeine.

By observing changes in planarian motility with exposure to guarana, caffeine and glucose, we determined that guarana seeds contain an additional stimulant over caffeine alone, and that glucose can support these tonic effects. We also provide evidence that guarana offers a short-term added benefit when combined with caffeine. Our data also suggest that the combination of guarana, caffeine and glucose provides a short-term stimulus at low concentrations.

## Materials and Methods

### Planarian Husbandry

Planarians (*Dugesia tigrina*) were purchased from Carolina Biological Supply Company (132954; Burlington, NC). This species is also referred to as *Girardia tigrina* [54]. Stocks were maintained in the dark using spring water (Poland Spring), which was often supplemented with 0.5 mM NaHCO_3_. Plastic food storage containers with lids ajar were used to house the specimens. Planarians were permitted to acclimate one week before feeding or experimentation. Organic, hard-boiled eggs were used as a food supply. Twice a week, a small amount of both egg white and yolk were chopped and placed into the containers. The planarians fed *ad libidum* for one to two hours. Planarians were then transferred into fresh containers and spring water (Poland Spring). The culture water was changed the following day to remove debris and to keep the water fresh.

### Planarian Locomotor Velocity (pLmV) Test

The planarian locomotor velocity (pLmV) test as established by R.B. Raffa and S.M. Rawls was adapted for this study [42, 43]. Briefly, planarians were habituated in their respective test substance or combination at the appropriate concentration for either two minutes or one hour. The worms were then transferred to the center of a 10 cm diameter Petri dish placed on grid paper, with lines spaced 0.5 cm apart, containing 20 mL of the same test solution. Planarian motility was monitored for 3 minutes by assessing the number of grid-lines crossed. Test concentrations were deemed as too high if the worms exhibited a coiling or convulsive behavior during the habituation period. Each experimental condition was tested a minimum of three times, and each test was typically done in triplicate if not more, so that the final pLmV data was assessed using at least nine worms for each condition. For each test, stock solutions of guarana extract, caffeine or glucose were diluted in spring water. Care was taken to conduct tests at different times of the day to account for different nascent or circadian activity rates. Two or three different trained experimenters conducted each set of tests. No detectable change in water quality, such as pH, resulted from the substances studied at the concentrations examined. Each worm, including control specimens, was used only once.

### Stock Solutions

All solutions were prepared using distilled water. Caffeine was purchased from Sigma Aldrich (C7731; St. Louis, MO) and was prepared as a 10 mM or 1 mM stock. D-glucose or dextrose was also obtained from Sigma Aldrich (D9434; St. Louis, MO) and was prepared as a 561 mM stock. The guarana used for this study was purchased online from Grass Hut Treasures (whole guarana seed powder; www.grasshuttreasures.com/guarana/guaranapowder.html). Guarana stocks were prepared based on the reported five percent caffeine content [19, 30]. To prepare 10 mM and 1 mM guarana extract, the appropriate mass of guarana seed powder was put into solution, mixed for several hours and then filtered twice to remove the insoluble material. The resulting guarana extracts were refrigerated and stored in foil-wrapped bottles to protect them from light.

### Statistics

All data were normalized to their respective controls and expressed as the mean +/- the standard deviation. Each data point was analyzed using a two-tailed, paired student’s T-test, and was deemed significant if the corresponding p-value was less than 0.05.

## Results

### Guarana Provides Additional Short-Term Stimulation over Caffeine Alone

The actual concentration of guarana present in energy drinks is typically not reported, but caffeine is considered the main stimulant present in the seeds [19]. Therefore, to prepare our guarana solutions we used published analyses of the caffeine content in guarana seeds as a guide. The assessed caffeine content of the guarana bean varied depending on the preparation and method of analysis—ranging from one to twelve percent—but was typically reported to be about five percent [19, 24, 30]. We then surveyed the caffeine content of energy drinks by looking at the information provided on the containers, and by using figs. available from online resources. This information was used to set concentration boundaries for our stimulant tests [55, 56]. These sources point to typical caffeine concentrations ranging from approximately 0.4 mM to just over 2 mM, so we chose a concentration range that would accommodate these, and allow us the freedom to study combinations of both purified caffeine and guarana while maintaining realistic caffeine concentrations. The chosen range covered 0.001 to 10 mM.

**Fig 1:**
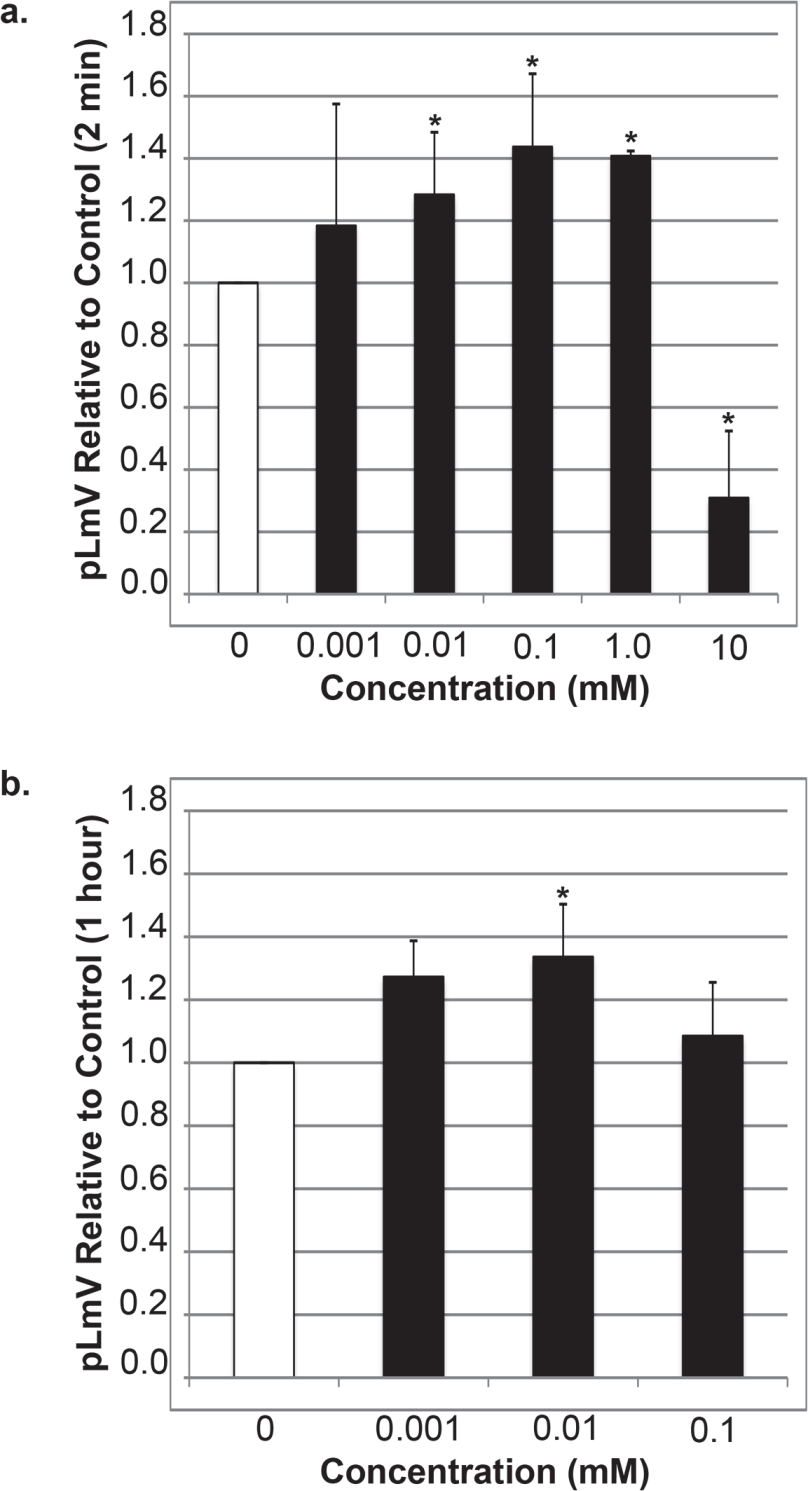
Planarian locomotor velocity (pLmV) increases in response to guarana alone. Shown are selected pLmV data relative to water-only controls for *Dugesia tigrina* exposed to varying concentrations of guarana extract. Planarians were habituated in their respective conditions for either 2 minutes (a) or 1 hour (b) before their pLmV activity was monitored. Planarian motility was monitored after the worms were placed in a Petri dish containing the appropriate concentration of guarana seed extract following the tested habituation period. The Petri dishes were placed over graph paper and the number of grid-lines crossed was monitored for three minutes. The white bar represents the normalized control pLmV, while the black bars indicate relative to control test pLmV values. Error bars indicate the standard deviation, while a * indicates a p-value of less than 0.05 relative to the water-only control.

The planarian locomotor velocity (pLmV) test was adapted to assess the stimulant properties of guarana [42, 43]. We began by testing a gauntlet of concentrations including 0.001, 0.003, 0.01, 0.03, 0.1, 0.3, 1.0, 3.0 and 10 mM and observing planarian motility after a two-minute incubation period in guarana seed extract (Fig 1A). From the tested concentrations, the most effective stimulation occurred above 0.003 mM, with significant stimulation occurring at 0.01 mM (p = 0.02), 0.1 mM (p = 0.008) and 1.0 mM (p = 0.006). 10 mM guarana seed extract proved inhibitory to planarian motility (Fig 1A) and caused the worms to coil repeatedly during the test. To determine if a longer exposure to guarana would provide an added stimulation, or allow the lower concentrations an opportunity to have an effect, we observed planarian motility after one hour in 0.001, 0.003, 0.01, 0.03 and 0.1 mM guarana seed extract (Fig 1B). As with the two-minute exposure, after one hour there was no significant stimulation of planarian motility at 0.001 and 0.003 mM. We still observed significantly increased activity at 0.01 mM (p = 0.009), but the effect was lost at 0.1 mM.

**Fig 2:**
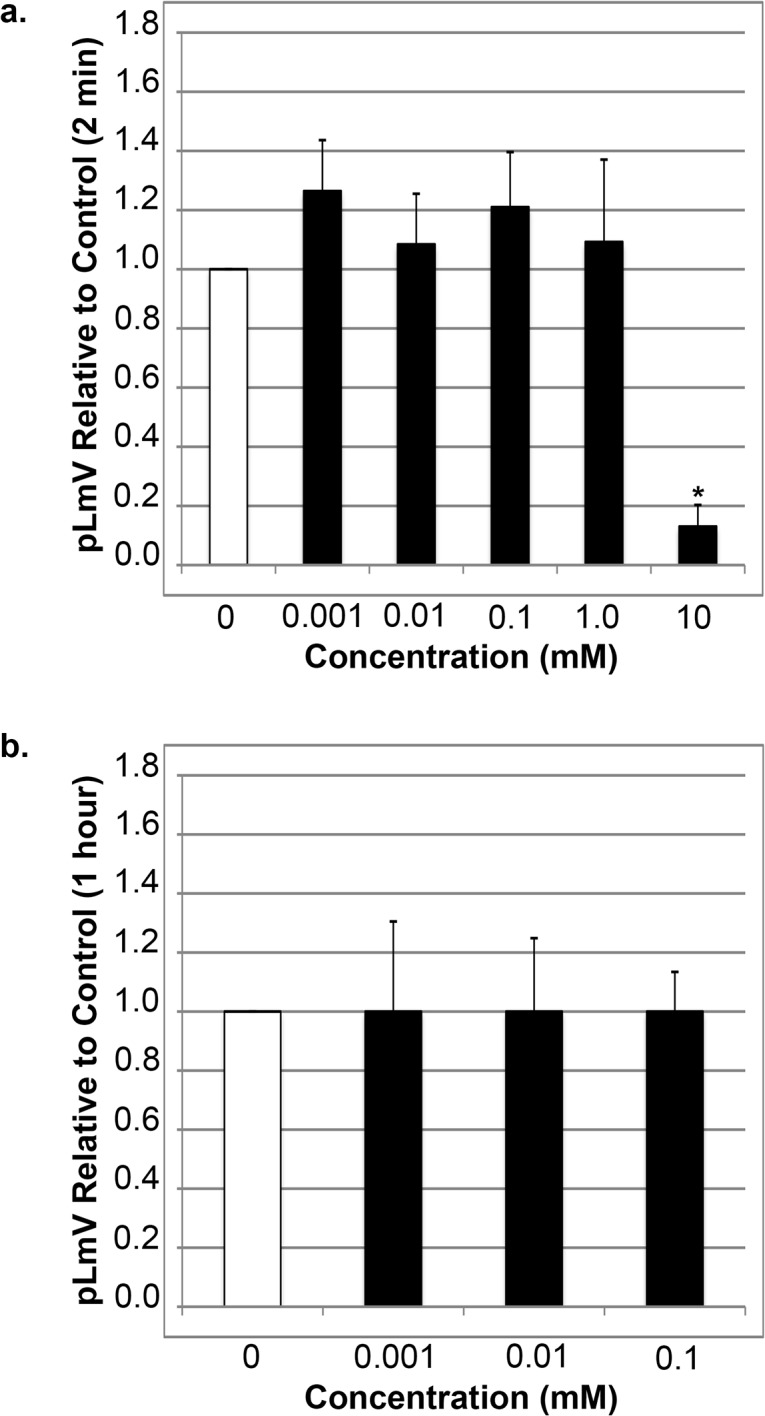
Caffeine alone does not support a significant increase in planarian locomotor velocity (pLmV). Shown are selected pLmV data relative to water-only controls for *Dugesia tigrina* exposed to varying concentrations of caffeine. Planarians were habituated in their respective conditions for either 2 minutes (a) or 1 hour (b) before their pLmV activity was monitored. Planarian motility was monitored after the worms were placed in a Petri dish containing the appropriate concentration of caffeine following the tested habituation period. The Petri dishes were placed over graph paper and the number of grid-lines crossed was monitored for three minutes. The white bar represents the normalized control pLmV, while the black bars indicate relative to control test pLmV values. Error bars indicate the standard deviation, while a * indicates a p-value of less than 0.05 relative to the water-only control.

Due to the fact that our results indicated that guarana does provide for significantly greater stimulation of planarian motility, we went on to test the effect of purified caffeine on pLmV. Since other investigators determined that caffeine does not provide a significant stimulation of planarian motility we aimed to use the system to assess whether guarana provides an added stimulus over caffeine alone [47, 50]. It was necessary, however, to determine if our system and choice of planarian species offered a comparable backdrop to these published reports. Again, we began by testing the planarian motility using a range of caffeine concentrations (0.001, 0.003, 0.01, 0.03, 0.1, 0.3, 1.0, 3.0 and 10 mM) after a two-minute period of exposure (Fig 2A). Although planarian motility was slightly elevated at concentrations between 0.003 mM and 3.0 mM, we did not observe any significant increase in overall planarian activity compared to the water only controls. However, because the calculated p-value for the pLmV using 0.001 mM caffeine was 0.05, suggesting a borderline significance, we tested if even lower concentrations of caffeine would produce a significant peak of activity. To this end, we examined the effect of both 0.0001 and 0.0003 mM caffeine, but still did not observe any significant increase in stimulation relative to the controls without caffeine (not shown). As with the guarana extract, 10 mM caffeine was inhibitory to planarian motility (Fig 2A) and resulted in sustained contractions during the assay period. These results were compared with a one-hour incubation time (Fig 2B). Just as with the guarana extract we examined 0.001, 0.003, 0.01, 0.03 and 0.1 mM, but again, caffeine alone did not cause a substantial rise in pLmV. These results confirmed that purified caffeine provides for a noticeable, albeit insignificant stimulation in planarian locomotion at the concentrations tested.

**Fig 3:**
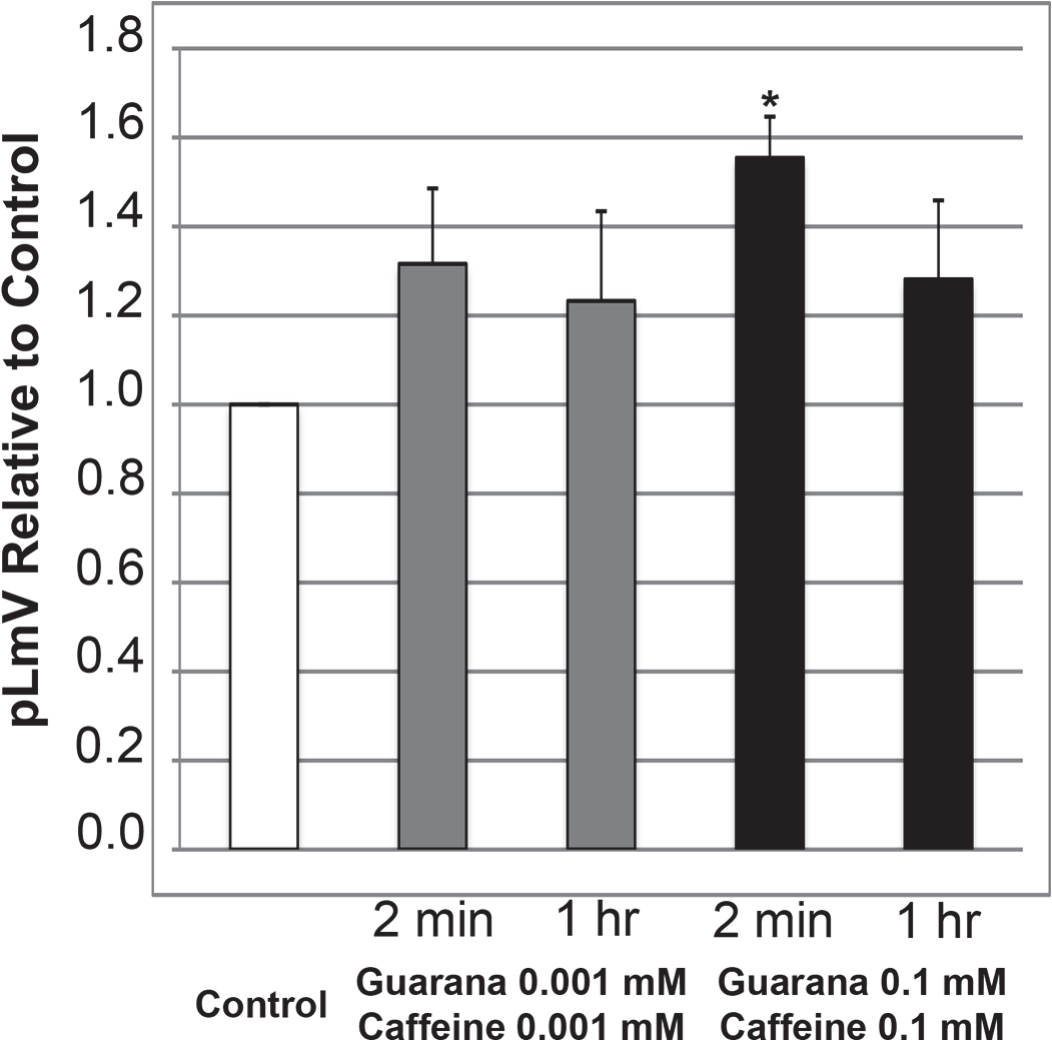
Guarana supports an additional short-term stimulation of planarian locomotor velocity (pLmV) over caffeine alone. Shown are pLmV data relative to water-only controls for *Dugesia tigrina* exposed a combination of 0.001 mM guarana seed extract and 0.001 mM caffeine (grey bars), or 0.1 mM guarana extract and 0.001 mM caffeine (black bars). Planarians were habituated in their respective conditions for either 2 minutes or 1 hour as indicated, before their pLmV activity was monitored. Planarian motility was monitored after the worms were placed in a Petri dish containing the appropriate concentrations of guarana seed extract and caffeine following the tested habituation period. The Petri dishes were placed over graph paper and the number of grid-lines crossed was monitored for three minutes. The white bar represents the normalized control pLmV, while the black bars indicate relative to control test pLmV values. Error bars indicate the standard deviation, while a * indicates a p-value of less than 0.05 relative to the water-only control.

To determine if an additional stimulant effect might be achievable by combining caffeine and guarana, we used solutions containing both low end and high end concentrations of caffeine and guarana seed extract and assessed planarian activity after exposure times lasting either two minutes or one hour (Fig 3). As 0.1 mM guarana seed extract provided the highest average relative pLmV over the concentration range tested, we chose this concentration for our high-end combination with caffeine. A caffeine concentration of 0.1 mM also provided the greatest average relative stimulation (calculated as 1.21) at the high-end compared to the water only control, despite being less than the 0.001 mM peak concentration—chosen for the low-end combinations—which had an average relative pLmV of 1.26 (Fig 2). A combination of 0.001 mM for both caffeine and guarana seed extract did not result in significantly elevated pLmV readings after either two minutes or one hour. Using a higher concentration of 0.1 mM for both solutions however, we did observe increased locomotor velocity after two minutes, but not after one hour (Fig 3). The average relative pLmV using the combined solution after the two-minute exposure was slightly higher than that observed for guarana extract alone, 1.55 (Fig 3) versus 1.44 (Fig 1), respectively. These two data sets were significantly different from each other with a p-value of 0.006. Our findings suggested that guarana provides an additional level of stimulation above that provided by caffeine alone.

### Low Glucose Concentrations Support Long-Term Guarana Stimulation

**Fig 4:**
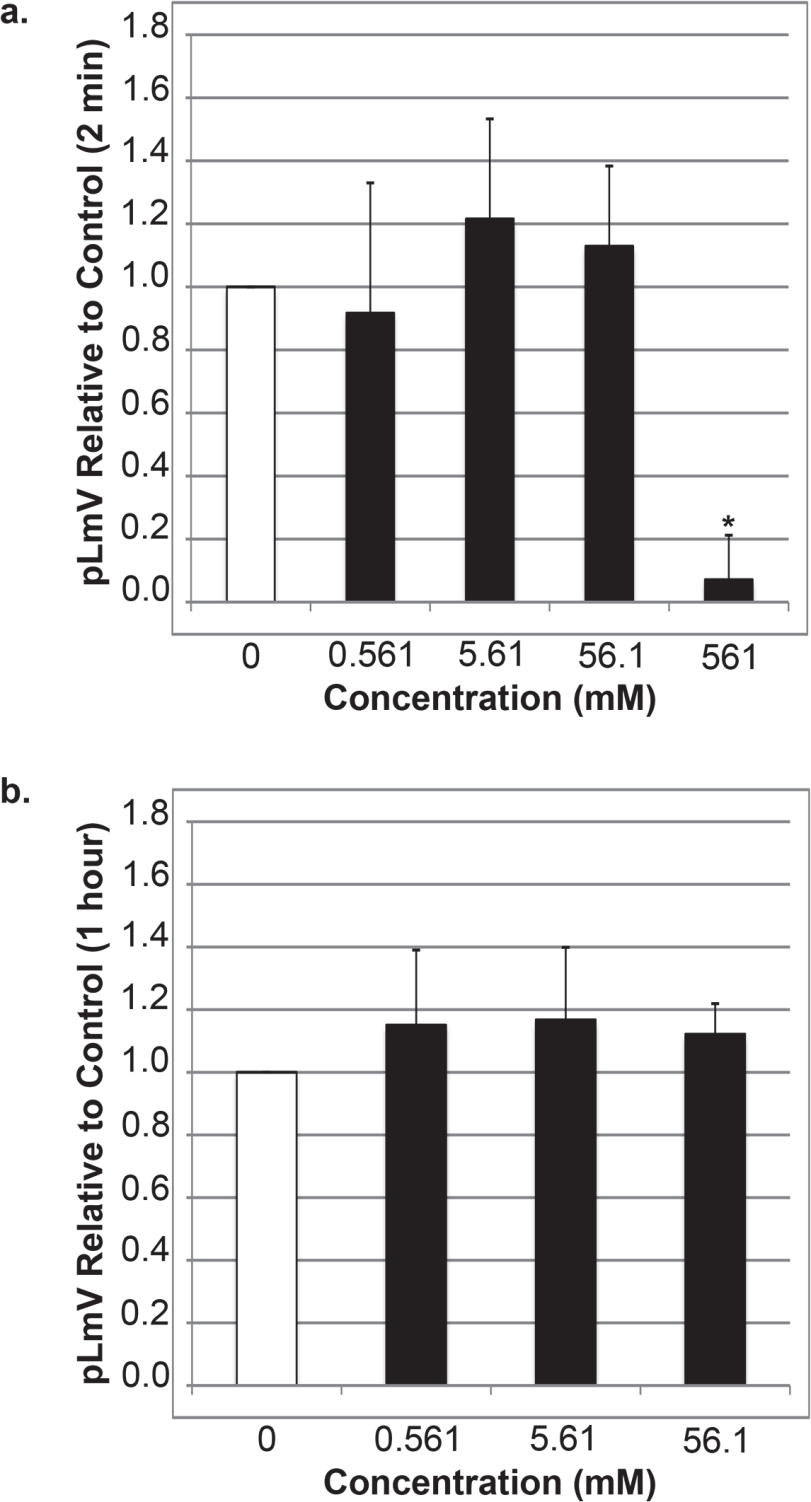
Glucose alone does not support a significant increase in planarian locomotor velocity (pLmV). Shown are selected pLmV data relative to water-only controls for *Dugesia tigrina* exposed to varying concentrations of glucose. Planarians were habituated in their respective conditions for either 2 minutes (a) or 1 hour (b) before their pLmV activity was monitored. Planarian motility was monitored after the worms were placed in a Petri dish containing the appropriate concentration of glucose following the tested habituation period. The Petri dishes were placed over graph paper and the number of grid-lines crossed was monitored for three minutes. The white bar represents the normalized control pLmV, while the black bars indicate relative to control test pLmV values. Error bars indicate the standard deviation, while a * indicates a p-value of less than 0.05 relative to the water-only control.

Since sugars are also a main ingredient in most energy drinks we next assessed how glucose influences guarana and caffeine stimulation. To begin, we determined how much sugar is present in these beverages by studying the ingredient list on the containers. Often the sugar content was not fully reported or was just listed as ‘sugar’, without providing details on exactly which sugar was considered. Further, if the type of sugar was listed, there were often different amounts reported for each—sucrose, fructose and glucose. Also ‘special blends’ were discovered to contain additional sugar without any concentration disclosure. We also assessed concentration ranges using online resources [55, 57]. We elected to use glucose as our sugar standard because it is the most easily metabolized sugar, and determined that sugar concentrations ranged from 290 to 730 mM. We then calculated the average sugar concentration listed on the beverage containers to be 561 mM, which we assumed to be glucose. We used that Fig. to prepare our standard stock solution. As with guarana and caffeine we conducted a pLmV study using glucose alone following exposure times of two minutes and one hour (Fig 4). The 561 mM concentration proved inhibitory to planarian locomotion as determined by repeated coiling behavior, and as such, we conducted our experiments with a series of glucose concentrations below that value (0.561, 1.683, 5.61, 16.83, 56.1, 168.3 and 561 mM). We did not detect any significant increase in pLmV using any of these concentrations after either a two-minute or one-hour exposure period. We did, however, detect a peak average relative stimulation of 1.22 at 5.61 mM after a habituation time of two minutes. This was significantly different from the average relative pLmV of 0.92 at 0.561 mM with a p-value of p = 6.0x10^-6^ (Fig 4A). In contrast, the average relative locomotor velocities for the glucose concentrations tested after a one-hour exposure to glucose (Fig 4B), did not result in any notable stimulation peaks above the water control despite a significant difference between 0.561 and 5.61 mM (average relative pLmV of 1.15 and 1.17, respectively; p = 0.011).

**Fig 5:**
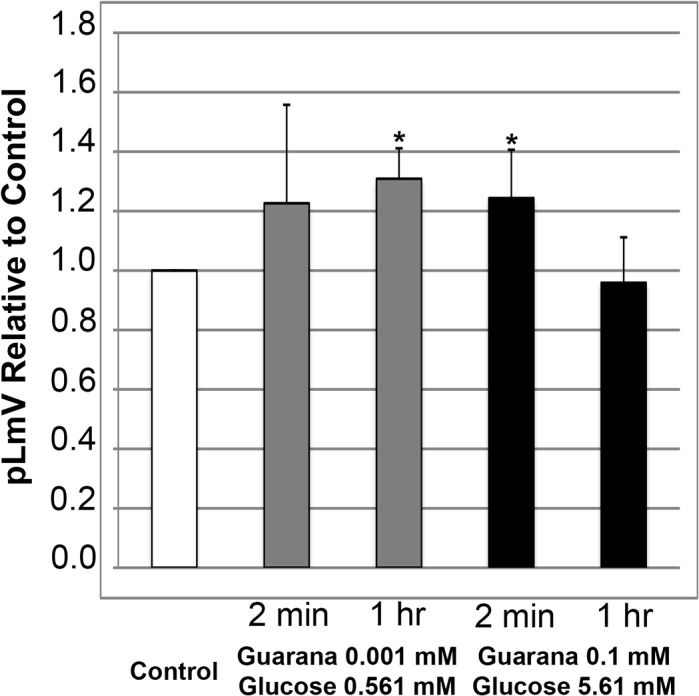
Glucose supports guarana stimulation of planarian locomotor velocity (pLmV). Shown are pLmV data relative to water-only controls for *Dugesia tigrina* exposed a combination of 0.001 mM guarana seed extract and 0.561 mM glucose (grey bars), or 0.1 mM guarana seed extract and 5.61 mM glucose (black bars). Planarians were habituated in their respective conditions for either 2 minutes or 1 hour as indicated, before their pLmV activity was monitored. Planarian motility was monitored after the worms were placed in a Petri dish containing the appropriate concentrations of guarana extract and glucose following the tested habituation period. The Petri dishes were placed over graph paper and the number of grid-lines crossed was monitored for three minutes. The white bar represents the normalized control pLmV, while the black bars indicate relative to control test pLmV values. Error bars indicate the standard deviation, while a * indicates a p-value of less than 0.05 relative to the water-only control.

We then assessed how the combination of guarana with glucose affected pLmV using low and high-end concentrations after two-minute and one-hour exposure times (Fig 5). At the low end, with a concentration of 0.001 mM guarana extract supplemented with 0.561 mM glucose, we observed a significant increase in motility after one hour (p = 1.5x10^-4^). Importantly, neither the guarana extract, nor glucose alone provided a significant stimulation at this time point at 0.001 mM, or 0.561 mM, respectively. This suggests that at low concentrations, glucose does provide a supportive effect to guarana stimulation over longer periods. In contrast, this effect was not apparent using a combined high-end concentration consisting of 0.1 mM guarana and 5.61 mM glucose, which was actually slightly repressive to planarian motility on average following a one-hour exposure period. The high-end concentration data, instead, resulted in a significant stimulation after two minutes (p = 0.032). It is notable that guarana did provide stimulation as a single reagent after two minutes at 0.1 mM with an average relative pLmV of 1.44 versus the water control (Fig 1A), and that with glucose that value actually dropped to a pLmV of 1.24 relative to the control (Fig 5). Statistical analysis indicated that the two-minute guarana single data at 0.1 mM and the combined high-end concentration data using guarana extract and glucose were significantly different from each other (p = 1.0x10^-7^).

**Fig 6:**
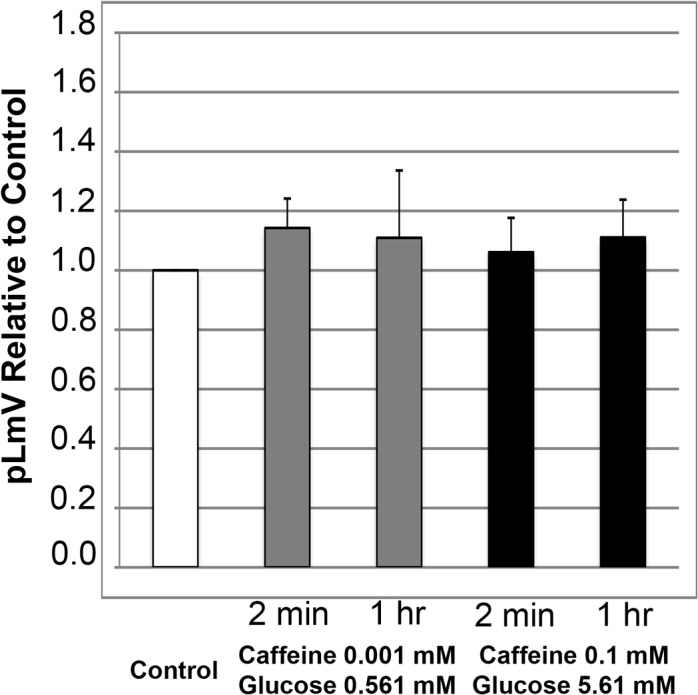
Glucose does not increase the effect of caffeine on planarian locomotor velocity (pLmV). Shown are pLmV data relative to water-only controls for *Dugesia tigrina* exposed a combination of 0.001 mM caffeine and 0.561 mM glucose (grey bars), or 0.1 mM caffeine and 5.61 mM glucose (black bars). Planarians were habituated in their respective conditions for either 2 minutes or 1 hour as indicated, before their pLmV activity was monitored. Planarian motility was monitored after the worms were placed in a Petri dish containing the appropriate concentrations of caffeine and glucose following the tested habituation period. The Petri dishes were placed over graph paper and the number of grid-lines crossed was monitored for three minutes. The white bar represents the normalized control pLmV, while the black bars indicate relative to control test pLmV values. Error bars indicate the standard deviation, while a * indicates a p-value of less than 0.05 relative to the water-only control.

In contrast, following with a complementary assessment of caffeine and glucose (Fig 6) we did not observe an increase in planarian locomotor velocity at either low or high-end concentration combinations for incubation periods of two minutes and one hour. These findings again highlighted a potential difference between guarana and caffeine in that the response to glucose was different between the two stimulants.

### Guarana in Combination with Glucose and Caffeine Provides a Short-Term Stimulus at Low Concentrations

**Fig 7:**
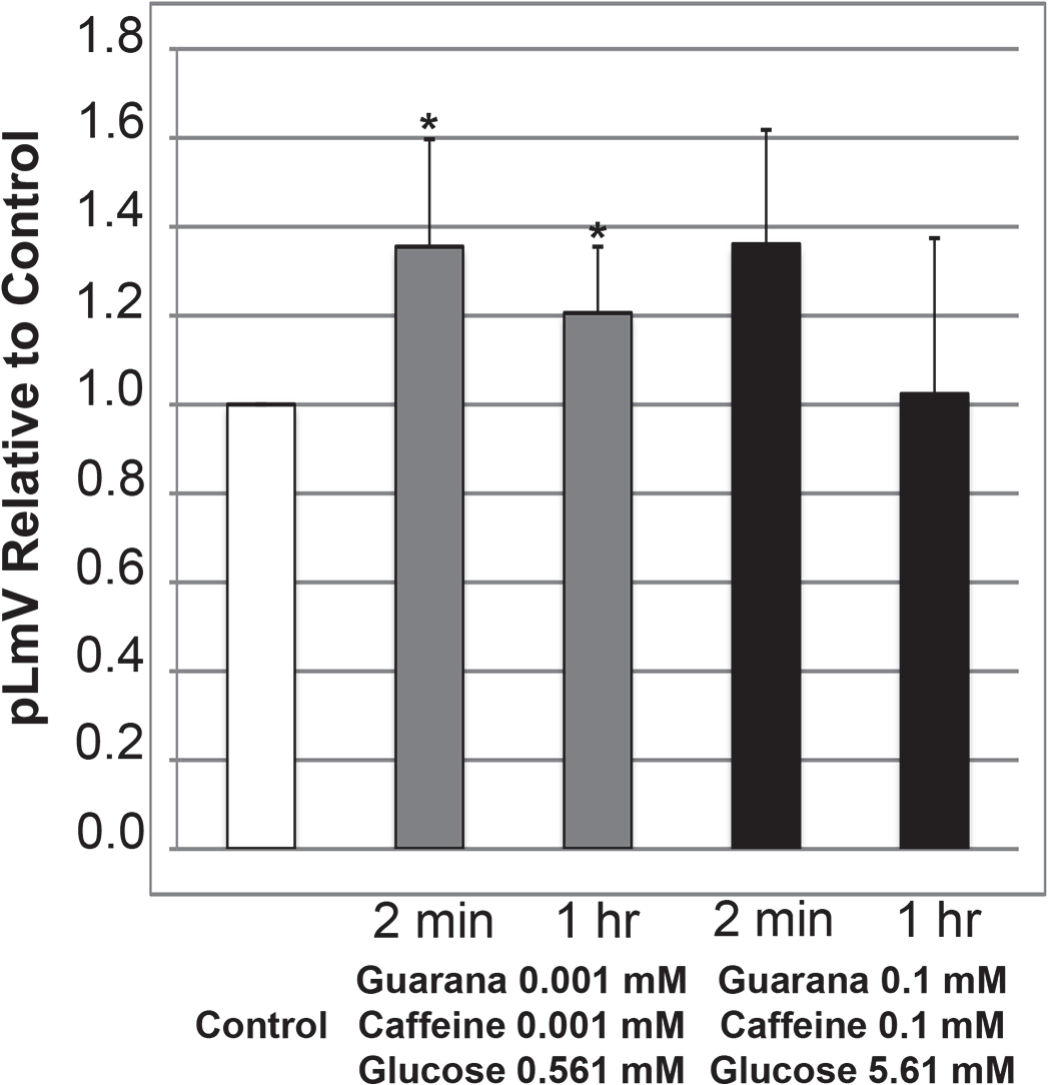
Guarana with glucose and caffeine supports planarian locomotor velocity (pLmV) at low-end concentrations. Shown are pLmV data relative to water-only controls for *Dugesia tigrina* exposed to a combination of 0.001 mM guarana seed extract, 0.001 mM caffeine and 0.561 mM glucose (grey bars), or 0.1 mM guarana seed extract, 0.1 mM caffeine and 5.61 mM glucose (black bars). Planarians were habituated in their respective conditions for either 2 minutes or 1 hour as indicated, before their pLmV activity was monitored. Planarian motility was monitored after the worms were placed in a Petri dish containing the appropriate concentrations of guarana extract, caffeine and glucose following the tested habituation period. The Petri dishes were placed over graph paper and the number of grid-lines crossed was monitored for three minutes. The white bar represents the normalized control pLmV, while the black bars indicate relative to control test pLmV values. Error bars indicate the standard deviation, while a * indicates a p-value of less than 0.05 relative to the water-only control.

Upon an assessment of locomotor velocity using guarana seed extract together with caffeine and glucose (Fig 7) we observed a slightly significant increase using low concentrations (0.001 mM for each guarana and caffeine, with 0.561 mM glucose) after only two minutes of habituation (p = 0.041). Importantly, we did not detect a low-concentration stimulation after two minutes with the double combinations of guarana seed extract and caffeine, guarana seed extract and glucose, or caffeine and glucose (Figs 3, 5 and 6, respectively). This suggests that some stimulant benefit is achievable through the combination of all three substances that might not be apparent otherwise at lower concentrations of any of the three substances alone, or in pairs, for the short-term. The significant low-concentration stimulus was maintained at one hour albeit at a lower level, with an average relative stimulation of 1.21 versus the 1.36 that was observed after two minutes (p = 0.016). But, we also observed a low-concentration average relative stimulus with guarana extract at 0.001 mM in combination with 0.561 mM glucose at one hour of 1.31 (Fig 5), which was greater than that observed with the addition of caffeine in the triple combination, suggesting that caffeine might not be contributing to the one-hour triple combination stimulation of pLmV at low concentrations.

In contrast, despite observing an improved pLmV relative to controls when planarians were exposed to a combination of guarana seed extract and caffeine at higher concentrations (0.1 mM of each; Fig 3), and a combination of guarana extract and glucose (0.1 mM guarana seed extract and 5.61 mM glucose; Fig 5) after only two minutes, we did not observe a significant added stimulation using higher concentrations of all three substances combined (0.1 mM for both guarana seed extract and caffeine, with 5.61 mM glucose) after the short-term two minute exposure. These data suggest that a short-term benefit is achieved with guarana, caffeine and glucose, but when combined at lower concentrations.

## Discussion

Energy drinks typically contain caffeine and sugars as their principal active ingredients. But many popular brands also contain blends of other substances that purportedly provide an added stimulus. One of these increasingly common additives is guarana. For this study we asked whether guarana had the potential to provide an added stimulant effect over caffeine alone using the planarian locomotor activity (pLmV) assay [42, 43]. Reports suggesting that planarian activity is not significantly affected by caffeine supported our choice of model system in that it allowed us to detect the tonic effects of guarana seed constituents separate from those provided by caffeine [47, 50]. Owing to the fact that caffeine is typically considered responsible for the stimulant properties of guarana, we used the reported five percent caffeine content of guarana seeds, and the typical range of caffeine concentrations reported for most energy drink formulations, as a starting point for our working guarana concentrations [19, 30, 55, 56].

When comparing a gauntlet of caffeine and guarana concentrations on planarian motility, it was indeed apparent that caffeine did not significantly increase pLmV at any concentration tested, while guarana had a positive effect at concentrations ranging from 0.01 to 1.0 mM (Figs 1 and 2). Notably, while not significantly different from the water controls, the average relative pLmV for each of the concentrations of caffeine above 0.1 mM following a two-minute habituation followed a decreasing trend, and significantly so above 1.0 mM, in that the p-values between these different caffeine concentrations were less than 0.05 (Fig 2A). This contrasted to the results for the guarana extract alone after two minutes, where we observed a peak stimulation using a concentration of 0.1 mM, but with a maintained significant stimulus over water controls at even a ten-fold higher concentration (Fig 1A). Had we chosen a less conservative caffeine content for our guarana seed powder, such as a high end estimate of 10 percent caffeine content—double our estimate—our high end concentration of 1.0 mM would be on the order of 2.0 mM caffeine and would have a much higher average relative pLmV compared to caffeine alone. Therefore, since the data for caffeine and the guarana extract do not follow the same overall pattern of activity, it further suggests that guarana provides another stimulant value separate from that of caffeine alone.

Inspecting the pLmV data collected after a one-hour habituation in caffeine alone there was no evidence of stimulation at any concentration tested indicating that even the trend for a low-level stimulus, while not significantly greater than the water control, was a short-term effect (Fig 2B). In contrast, the post one-hour data collected for guarana seed extract-exposed pLmV showed a significant stimulus at 0.01 mM, but not at 0.1 mM (Fig 1B). These guarana findings suggested that a longer-term stimulus could be maintained at lower concentrations, providing further evidence in support of a difference between guarana and caffeine stimulation. In general, however, we suggest that the one-hour data for both caffeine and guarana indicate that a tolerance-type of response occurs with length of exposure to higher concentrations of these stimulants. Importantly, others have observed that planarians respond best to stimulants within the first five minutes of exposure, which is reflected in our findings as well [50].

Our locomotor data combining selected low and high concentrations of guarana seed extract and caffeine (Fig 3) suggested that guarana provides an additional stimulation above that provided by caffeine alone. Neither guarana seed extract nor caffeine alone provided a detectable increase in pLmV as a single stimulant at the low concentration examined (0.001 mM, Figs 1 and 2, respectively) and this did not change when the substances were combined (Fig 3). But, when the higher concentrations of 0.1 mM were combined, an increase in the relative average motility was observed following a short-term, two-minute, exposure time (Fig 3). This average relative pLmV was augmented over that observed for guarana extract alone pointing to an additional short-term stimulation through the combination of higher concentrations of guarana and caffeine. Furthermore, since such an additive effect was not observed by simply increasing the concentration of caffeine (Fig 2), the additional stimulation provided by guarana seed extract might be provided by some other water soluble ingredient in the seeds that functions using a metabolic pathway separate from that used by caffeine. Significantly, another group examining the effects of guarana on mice also observed increases in activity that likely operated using a different mechanism than that used by caffeine [20]. While supporting our hypothesis that guarana contains stimulant properties independent of caffeine, this corroborating result also supports the use of the planarian for studies implicating stimulant effects in mammalian systems.

The stimulant effect of caffeine is suggested to be mediated by a number of mechanisms including the intracellular mobilization of calcium, the inhibition of phosphodiesterases, the binding of caffeine to benzodiazepine receptors, and antagonism at the level of adenosine receptors [58, 59]. While the caffeine in guarana likely works by these mechanisms, other constituents possibly function using different pathways. In addition to caffeine, guarana seeds are known to harbor a number of other possible stimulants such as catachins, tannins and other alkaloids such as theophylline and theobromine [19, 22, 30, 60]. At present, little information is available favoring one of these ingredients over the others.

We expanded our work to consider the effect of sugar on pLmV in conjunction with both caffeine and our guarana seed extract. The specific sugars in energy drink varieties vary, and as such we chose to work with D-glucose, or dextrose in our study. Not all producers of energy drinks disclose complete concentrations of sugars in their formulations, so we calculated an average concentration to use in our experiments. We arrived at this value by averaging the amount of sugar disclosed on the few containers that gave some details of the total sugars they contained. We compared this amount with those of estimates of total sugars in energy drinks discovered online, which were reflected in our chosen stock concentration [55, 57]. While we observed that glucose alone did not offer an apparent stimulation of planarian motility (Fig 4), it did support guarana stimulation at low-end concentrations for a longer time (Fig 5). This was particularly interesting in that neither glucose alone (Fig 4B) nor guarana seed extract alone (Fig 1B) resulted in an increased pLmV after the one-hour habituation at these concentrations. We also observed a significant short-term relative average stimulation with the combined high-end concentrations of glucose and guarana seed extract (Fig 5), but since the guarana seed extract alone resulted in a significantly augmented pLmV (Fig 1) after the same time period, it was not possible to determine if glucose further intensified this effect using our system. But, our data do suggest that at low concentrations, glucose does provide a supportive effect to guarana stimulation over longer periods. These data are in line with published reports suggesting that glucose can support the effect of stimulants in the planarian model [48]. Conversely, our combinations of glucose and caffeine did not result in an increase of planarian motility at either time-point examined (Fig 6) providing further evidence of stimulant properties in guarana that are indeed different from those of caffeine.

Exposing planarians to guarana seed extract, caffeine and glucose together resulted in augmented planarian motility after both short and long-term stimulation, but only with our low concentration combination (Fig 7). However, since our low concentration of guarana seed extract coupled with our low concentration of glucose also offered a sustained significant stimulation after the one-hour incubation, we cannot conclude that caffeine had an additional effect in this situation. On the other hand, the triple combination was the only one tested that resulted an increased pLmV after only two minutes of stimulant exposure. Significantly, these data provide evidence that low concentrations of guarana, caffeine and glucose in combination are sufficient to provide a short-term stimulus.

We reiterate that since both caffeine and glucose provide only a low level of stimulation in the planarian model, we were able to detect the possibility of other stimulating substances within a guarana seed extract and assess whether caffeine and glucose can augment those effects. Future studies will be required to identify the actual additional stimulant contained in guarana [19]. But, our overall impression from this work is that guarana does offer supplementary stimulation over caffeine, and that both caffeine and glucose can change the nature of this stimulation. It is apparent that in order to fully appreciate how energy drinks affect physiology, it is important to consider the combination of the substituents they contain in that these substances may behave differently when mixed together in the same formulation. Indeed, others have also put forward the idea of studying energy drink components in combination using human models [9, 18, 25, 61], but given the complexities in controlling human dietary habits, particularly when caffeine is involved, we suggest that the planarian system offers a straightforward first approach to examine these complex interactions. It is also noteworthy that our observations made when combining guarana seed extract, caffeine and glucose suggested a short-term benefit in low concentrations counters the general perception that ‘more is more’ in energy drink formulations. Further research is required, but our preliminary findings using the planarian model suggest that lower doses of stimulants can work together to provide a short-term stimulant effect, and that combining greater amounts of these ingredients might not provide a long-term benefit.
